# Health systems stewards and health systems researchers: a critical partnership for malaria elimination

**DOI:** 10.1186/1475-2875-11-S1-O13

**Published:** 2012-10-15

**Authors:** Don de Savignv

**Affiliations:** 1 Swiss Tropical and Public Health Institute, University of Basel, Switzerland

## 

The Global Malaria Action Plan places scaled-up effective malaria control as a necessary prerequisite for malaria elimination. Effective and sustained malaria control depends on vastly strengthened health systems in malaria endemic countries. However definitions of health system strengthening, and the means to achieve it are still lacking. Emerging approaches of system-wide thinking that applies systems thinking tools in implementation science can open new possibilities to accelerate system strengthening with a particular focus on the systems effectiveness of malaria surveillance and control interventions. This presentation will discuss how tipping point revolutions in health systems could be harnessed to strengthen health systems to become malaria elimination ready. These include social network analysis to better manage stakeholder partnerships that bring malaria control and implementation research closer together; new concepts in governance of health systems to improve both system design and systems management; new approaches to diagnose system effectiveness bottlenecks and target system-level interventions; and modern mHealth strategies to improve procurement and supply chains as well as surveillance as an intervention in real time. For rapid progress across these dimensions, closer and better managed partnership among health system managers, health systems researchers and implementation scientists is essential.

